# Impact of Adding a Decision Aid to Patient Education in Adults with Asthma: A Randomized Clinical Trial

**DOI:** 10.1371/journal.pone.0170055

**Published:** 2017-01-20

**Authors:** Myriam E. Gagné, France Légaré, Jocelyne Moisan, Louis-Philippe Boulet

**Affiliations:** 1 Knowledge Translation, Education and Prevention Chair in Respiratory and Cardiovascular Health, Laval University, Quebec City, QC, Canada; 2 Quebec Heart and Lung Institute-Laval University, Quebec City, QC, Canada; 3 Canada Research Chair in Implementation of Shared Decision Making in Primary Care, Laval University, Quebec City, QC, Canada; 4 CHU de Quebec Research Center, Population Health and Optimal Health Practices Research Unit, Quebec City, QC, Canada; 5 Chair on Adherence to Treatments, Laval University, Quebec City, QC, Canada; Universite de Bretagne Occidentale, FRANCE

## Abstract

**Background:**

Not providing adequate patient education interventions to asthma patients remains a major care gap. To help asthma patients and caregivers discuss inhaled controller medication use, our team has previously developed a decision aid (DA). We sought to assess whether adding this DA to education interventions improved knowledge, decisional conflict, and asthma control among adults with asthma.

**Methods:**

A parallel clinical trial (NCT02516449). We recruited adults with asthma, aged 18 to 65 years, prescribed inhaled controller medication to optimize asthma control. Educators randomly allocated participants either to the education + DA or to the education group. At baseline and two-month follow-up, we measured asthma knowledge (primary outcome) with a validated self-administered questionnaire (score –37 to +37). Secondary outcomes included decisional conflict and asthma control. Blinded assessors collected data. Between the two time points, the within- and between-group changes were estimated by generalized linear mixed models.

**Results:**

Fifty-one participants (response rate: 53%; age: 44 ± 13 years; women: n = 32) were randomized either to the education + DA group (n = 26) or to the education group (n = 25), and included in statistical analyses. Between baseline and follow-up, mean [95% CI] knowledge scores increased from 21.5 [19.9–23.2] to 25.1 [23.1–27.0] in the education + DA group (*P* = 0.0002) and from 24.0 [22.3–25.7] to 26.0 [24.0–28.0] in the education group (*P* = 0.0298). In both of the groups, decisional conflict and asthma control improved. There were no differences between groups.

**Conclusions:**

Education improved knowledge, decisional conflict, and asthma control whether the DA was added or not.

## 1 Introduction

Asthma is a chronic respiratory disease that affects 300 million people worldwide [1]. It is associated with high clinical and economic burden, since it not only increases resource utilization [2–4] and lost school days and workdays [2–4], but it also has a high cost of around €1,600 per patient, from a societal perspective [3].

To prevent symptoms and exacerbations–and thereby reduce asthma-related morbidity–, adults with mild to severe asthma must adhere to a highly effective controller pharmacotherapy regimen, use their inhalers correctly, avoid or reduce exposure to indoor allergens and environmental tobacco smoke exposure, quit smoking, monitor their control of asthma, use their written action plan when experiencing worsening symptoms, and attend their follow-up appointments [5]. But to play such an active role in the management of their condition, persons with asthma first need to acquire the knowledge, skills, and attitudes that are necessary to achieve their optimal health potential [5, 6].

Patient education is an active process by which health care professionals guide and support patients in applying what they have learned and thus adhering to their treatment plan [6]. It is known to improve quality of life and asthma control in adults with asthma [7, 8], and to also be cost-effective [9]. In addition to giving patients factual and unbiased information, patient education tailors teaching to the patients’ needs and requires identification and acknowledgment of their concerns, as part of a patient-centered care approach [6].

Shared decision making integrates patient-centered concepts [10]. It has been advocated as a means to foster the collaborative participation between patients and clinicians in health care decisions [11], and described as the pinnacle of optimal patient care [12]. In asthma, a shared decision making intervention resulted in a significant improvement in asthma medication adherence, but did not rely on the use of a decision aid (DA) [13].

DAs support health care professionals in conveying evidence-based information about treatment options and patients in communicating their values and preferences regarding those options [14–16]. Evidence from systematic reviews indicates that DAs could be effective in implementing shared decision making in clinical practice [16, 17]. When compared to standard care interventions, DAs that target patients with conditions or chronic diseases other than asthma have a positive and significant impact on decision quality attributes, defined as improved knowledge and lowered decisional conflict [16] (the state of being uncomfortable about a decision, which is driven by modifiable factors such as lack of knowledge, unclear values, and inadequate support [18]). A high-quality decision, in turn, might affect behavioral and health outcomes [19].

In the specific context of chronic disease care, DAs have been shown to increase the use of options that are beneficial to the patients, but are underused [13, 20]. Inhaled controller medications, especially inhaled corticosteroids, with or without long-acting β_2_-agonists, have been described as a beneficial asthma treatment, because they improve the patients’ quality of life and optimize asthma control in adults with asthma [21]. Although they are considered as the cornerstone of the asthma treatment regimen [5], results from systematic reviews indicate that they remain underused [22–25]. In turn, inhaled controller medication underuse has been associated with a high economic burden, because it leads to poor asthma control [22, 24], and increases hospitalizations [22, 25] and costs [22, 24].

In asthma, the underuse of inhaled controller medications is somewhat driven by the patients’ perception of medication safety [26]. Many patients have concerns about using inhaled medications that they might not have fully discussed with a health care professional [27], and current clinical practice guidelines are not necessarily geared at taking into account what patients value the most [28, 29]. In this context, our team developed, to the best of our knowledge, the first DA for adults with asthma considering the use of inhaled corticosteroids, with or without long-acting β_2_-agonists, to optimize asthma control [30]. In the present study, we sought to assess whether or not adding our DA to education could improve asthma knowledge in adults with asthma. In addition, we explored whether it could lessen decisional conflict and enhance the appropriate use of asthma pharmacotherapy as well as asthma control among adults with asthma.

## 2 Materials and Methods

### 2.1 Trial design

The study was designed as a prospective two-month randomized controlled parallel group trial (allocation ratio 1:1). It was approved by the Institutional Ethics Committee of the Quebec Heart and Lung Institute on November 5^th^, 2012 (approval number: CER20858). The recruitment period lasted from March 12^th^, 2013 to September 9^th^, 2013. Both the period of follow-up and the trial ended on November 15^th^, 2013. All participants gave written informed consent. Due to time constraints, the study was registered on *ClinicalTrials*.*gov* after recruitment of the first study participant, but before data analysis (Clinical Trial Registry Number: NCT02516449). The authors confirm that there were no ongoing and related trials for this intervention, otherwise they would have also been registered.

### 2.2 Participants

A convenience sample of participants was recruited when attending the Quebec Heart and Lung Institute, a tertiary care center in Quebec City, Quebec, Canada. Eligible participants were (1) between the ages of 18 and 65, (2) diagnosed with mild to severe asthma, and (3) prescribed inhaled corticosteroids, either alone or in combination with long-acting β_2_-agonists, to optimize asthma control. The asthma diagnosis was either based upon objective measures of lung function [31] or made by a pulmonologist.

Individuals were not eligible to participate in the study if they had participated in the development of the DA [32] or if they were provided patient education in the six preceding months. To ensure that individuals suffering from chronic obstructive pulmonary disease (COPD) would not be included, we excluded persons aged ≥40 years, with prebronchodilator forced expiratory volume in one second (FEV_1_) <80% of predicted value, or with smoking history of >10 pack-years.

Data were collected on the recruitment site.

### 2.3 Interventions

#### 2.3.1 Experimental group: Education + DA

Participants in the experimental group as well as in the control group (see Section 2.3.2) met a certified asthma educator (hereafter referred to as an educator) from the Quebec Asthma and COPD Network [33] for patient education (see details of the education component below, Section 2.3.2). Prior to patient education, participants of the experimental group read and filled the DA.

The DA, available at www.coeurpoumons.ca, considered the following index decision: *Which option would be best for me*, *while considering inhaled corticosteroids*, *with or without long-acting β*_*2*_*-agonists*, *to optimize asthma control*? Its development process was described elsewhere [30]. The DA was designed according to the four-step structure of the Ottawa Personal Decision Guide [34, 35]. The first step presented information on asthma physiopathology, the role of inhaled corticosteroids and long-acting β_2_-agonists, and the two options that were discussed: to take (option #1) or not to take (option #2) the prescribed inhaled controller treatment to optimize asthma control. The second step described the positive and negative features of taking the medication and participants compared and weighed the expected benefits and risks of using the prescribed treatment. The third step identified participants’ decisional making needs, using the French version [36] of the 4-item SURE test [37]. In the fourth and final step, participants indicated whether or not they would take the prescribed inhaled controller medication or if they were unsure about taking the treatment.

#### 2.3.2 Control group: Education alone

As required by the *Canadian Thoracic Society Asthma Management Continuum* [31], educators delivered patient education interventions. Participants were provided with information on asthma diagnosis, physiopathology, and environmental control. They were explained the difference between reliever and controller medications and why the latter should be taken regularly. Participants were advised of medication safety as well as of the potential side effects of pharmacological treatments. To ensure optimal drug delivery, they were taught about the correct inhalation technique. Participants were also provided with an individualized written action plan.

During the intervention, educators elicited patients’ illness beliefs and concerns by asking patients open-ended questions, and provided them with feedback. Moreover, patient education extended beyond the verbal exchange of information and was tailored to the patient’ specific context. As a supplementation of verbal information, pictograms and kinaesthetic materials were used.

### 2.4 Outcomes

Knowledge of asthma was defined as the primary outcome whereas secondary outcomes included decisional conflict, appropriate use of pharmacotherapy, and asthma control.

#### 2.4.1 Knowledge of asthma

Knowledge of asthma was measured by the *Questionnaire de connaissances sur l’asthme de langue française* (QCALF). The QCALF is self-administered French instrument that was shown to have good reliability and reproducibility [38]. It evaluates four domains of asthma knowledge: biomedical, asthma severity, general knowledge and treatment [38]. The questionnaire comprised 37 items with response options labeled true, false, and don’t know. Right answers were scored +1, wrong answers -1, and don’t know answers 0. Item scores were summed. The total QCALF score ranged from -37 to +37.

#### 2.4.2 Decisional conflict

Decisional conflict was measured by the French version [39] of the *Decisional Conflict Scale* (DCS), a self-administered instrument that was shown reliable and responsive to change [18, 40, 41]. Using a five-point Likert scale (0 = strongly agree; 4 = strongly disagree), 16 items assessed five dimensions of decisional conflict: informed, values clarity, support, uncertainty, and effective decision. Participants’ responses were summed, divided by 16, and multiplied by 25. The total DCS score ranged from 0 to 100. A score ≥37.5 was suggestive of meaningful decisional conflict or delayed decision implementation [18].

#### 2.4.3 Appropriate use of asthma pharmacotherapy

The appropriate use of asthma pharmacotherapy was evaluated using a four-item face-to-face interviewer-administered questionnaire [42]. For participants to be considered as appropriate users of asthma drugs, they needed to meet eleven hierarchical criteria, which included using their controller medications for the same number of times every day and at an adequate frequency [42].

#### 2.4.4 Asthma control

Asthma control was quantified using the clinical and physiological subscales of the validated *Asthma Control Scoring System* (ACSS) [43]. The clinical subscale was interviewer-administered. To measure the ACSS physiological parameter, a spirometry was performed using Medisoft Exp’air Micro 5000 (Roxon medi-tech, St-Léonard, Quebec, Canada), according to the American Thoracic Society criteria [44]. The percentage of predicted value of FEV_1_ was derived from the Global Lung Initiative 2012 [45]. The clinical and physiological parameter scores were averaged. The total ACSS score ranged from 20 to 100. A score ≥80% was indicative of asthma control [46].

Using a standardized self-administered form, sociodemographic and clinical data about the participants’ highest attained level of education, year of asthma diagnosis, allergy, smoking status, and respiratory tract infections were collected. Gender, date of birth, height and weight, were abstracted from the participants’ medical charts, in addition to the prescribed daily dose inhaled corticosteroids, which defined asthma severity [31].

Measurements were undertaken at baseline, prior to intervention allocation, and at 2-month follow-up. Self-administered questionnaires were filled in first, followed by face-to-face interviewer-administered questionnaires. Spirometry was performed last.

### 2.5 Sample size

We calculated the sample size on the basis that the improvement in asthma knowledge scores would be greater in the education + DA group than in the education group [16]. We used the SAS generalized estimating equation (GEE) macro for controlled clinical trials with repeated measurements on the same individuals to estimate the sample size [47]. Based on a previous study [48], we assumed that asthma patients would be found to have an asthma knowledge score of 26.5 (standard deviation = 4.3) on the QCALF at baseline. We expected our control group participants to have a 5-point improvement in their mean asthma knowledge score over time, and the experimental group to have a 10-point improvement. Put another way, we expected a large effect size of 1.16 [49]. As a result, we calculated that a sample size of 24 participants per group was required to detect a group-by-time interaction estimate of five point, with type I error = 0.05, type II error = 0.20 (80% power), and a two-sided test. Additionally, this was considered feasible, as the present study was carried out as part of the Master’s thesis of the first author (MG).

### 2.6 Randomization

A statistician generated a random allocation sequence of block size of four using a computer software program. The study coordinator enrolled participants. Educators assigned participants to interventions using sequentially numbered, opaque, sealed and equally weighted envelopes.

### 2.7 Blinding

After assignment to interventions, only the study coordinator, who assessed the outcomes, was blinded.

### 2.8 Statistical analyses

Data were analyzed by intention-to-treat [50]. We used generalized linear mixed models that accounted for repeated measurements [51] to assess the impact of adding the DA to patient education. All models included a group variable (education + DA versus education alone), a time variable (two-month follow-up versus baseline), and a group-by-time interaction term. With the repeated measurements, we assumed a specified form of covariance structure among the two visits in which estimates and standard errors were based on a restricted likelihood function given the observed data (REML). Using an unstructured covariance matrix, this specification permitted to handle missing values at the follow-up visit [52]. Models estimated means or prevalence with corresponding 95% confidence intervals, as well as within and between-group differences in means and prevalence ratios. The estimate of the group-by-time interaction term was of primary interest [51]. To fulfill model assumptions, decisional conflict scores underwent a natural log transformations [53]. To facilitate interpretation, means and 95% confidence intervals were back-transformed on their original scale [53]. Determinants of decisional conflict (knowledge [54]), appropriate use of pharmacotherapy (knowledge [42], age [55]), and asthma control (knowledge, body mass index, age, allergy and respiratory tract infections [56]) identified *a priori* were not included in statistical models since they did not result in a >10% change in the mean differences or prevalence ratios [57]. SAS 9.4 (SAS Institute Inc., Cary, North Carolina, USA) was used to perform all statistical analyses. A two-tailed *P*-value <0.05 was considered indicative of statistical significance.

## 3 Results

### 3.1 Participant flow

**Fig 1:**
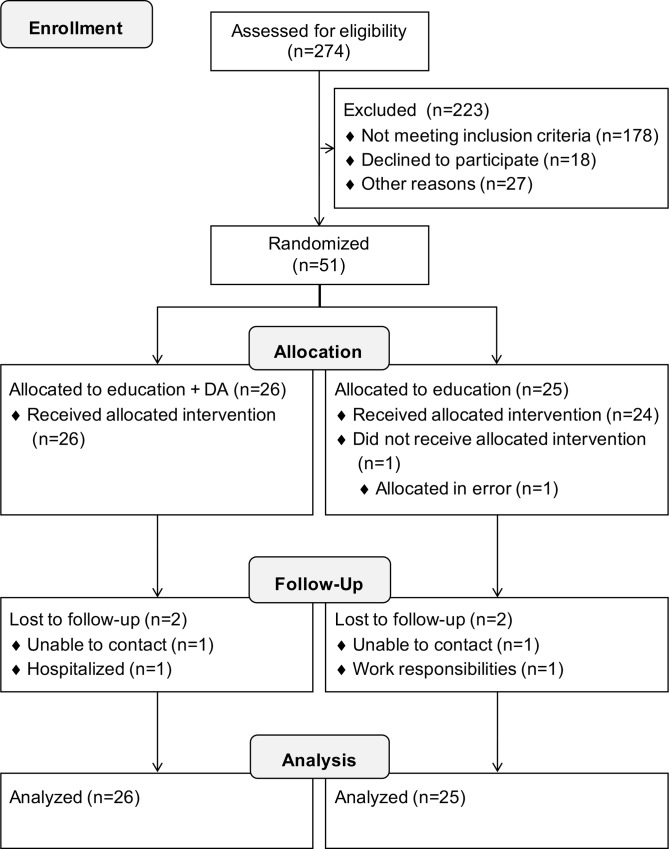
Flow of participants through the study. **CONSORT flow diagram.** Adapted from the CONSORT 2010 Flow Diagram [58].

Fig 1 illustrates the flow of participants through each stage of the study. Two hundred seventy-four persons were screened for eligibility. Out of the 96 eligible individuals, 51 participants were enrolled (response rate of 53%). Twenty-six participants were randomly allocated to the education + DA group and 25 to the education group. One participant did not receive the allocated intervention. Forty-eight participants completed the study (education + DA group: n = 25; education group: n = 23). One participant of the education + DA group, who was hospitalized during the follow-up visit for an event unrelated to the study, was considered as lost to follow-up. All participants were included in data analysis.

### 3.2 Recruitment

The recruitment period lasted from March 12^th^, 2013 to September 9^th^, 2013. Both the period of follow-up and the trial ended on November 15^th^, 2013.

### 3.3 Baseline data

Baseline demographic and clinical characteristics of the study group participants are presented in Table 1. Participants (women, n = 32) were aged [mean ± standard deviation] 44 ± 13 years. About half of the participants had a university diploma (n = 23). The mean asthma duration was 22 years. Most of the participants were suffering from moderate asthma (n = 34) and were non-smokers (n = 35).

**Table 1:** Baseline demographic and clinical characteristics of participants (*N* = 51).

		Education + DA*N* = 26	Education*N* = 25
**Gender**			
	Men	7	12
	Women	19	13
**Age (years)**		46 ± 13	41 ± 13
**Body mass index**			
	Under- and normal weight (BMI <25 kg/m^2^)	8	10
	Overweight (25≤ BMI <30 kg/m^2^)	11	8
	Obesity (BMI ≥30 kg/m^2^)	7	7
**Highest level of education attained**			
	<University	15	13
	University completed	11	12
**Spirometry**			
	FEV_1_ (% predicted)	79.3 ± 19.6	84.9 ± 17.0
	FVC (% predicted)	95.4 ± 14.7	89.9 ± 17.5
**Asthma severity**			
	Mild	4	1
	Moderate	16	18
	Severe	6	6
**Number of asthma medications**			
	1–2	23	19
	3–6	3	6
**Duration of asthma (years)**^**a**^		22 [0–52]	9 [0–53]
**Smoking status**			
	No smoker	19	16
	Ex-smoker	6	8
	Current smoke	1	1
**Self-reported allergy**		21^a^	24
**Self-reported respiratory tract infections**		8^b^	9

Data are expressed either as n, mean ± standard deviation, or median [range]. FEV_1_: Forced expiratory volume in one second; FVC: Forced vital capacity.

^a^Missing data: n = 1

^b^Missing data: n = 2

### 3.4 Outcomes and estimation

Between baseline and follow-up, mean [95% CI] knowledge scores significantly increased from 21.5 [19.9–23.2] to 25.1 [23.1–27.0] in the education + DA group (*P* = 0.0002) and from 24.0 [22.3–25.7] to 26.0 [24.0–28.0] in the education group (*P* = 0.0298). As shown in Table 2, these improvements were not different between groups (*P* = 0.24). Between the two time points, decisional conflict scores decreased from 12.3 [7.7–19.7] to 4.2 [2.4–7.3] in the education + DA group (*P* = 0.0073) and from 15.8 [9.7–25.5] to 6.7 [3.8–11.8] in the education group (*P* = 0.0093). The proportion of participants who used their asthma drugs appropriately increase from 0.42 [0.26–0.70] to 0.57 [0.38–0.85] in the experimental group (*P* = 0.25) and from 0.24 [0.12–0.48] to 0.40 [0.25–0.65] in the control group (*P* = 0.14). Asthma control scores were higher in both of the groups at two-month follow-up, compared to baseline (education + DA: baseline: 73.2 [66.1–80.2] and follow-up: 81.6 [75.7–87.6], *P* = 0.0063; education: baseline: 74.1 [66.9–81.3] and follow up: 83.4 [77.3–89.5], *P* = 0.0036). As shown in Table 2, changes in baseline decisional conflict and asthma control scores, as well as in the proportions of appropriate users of asthma drugs, did not appear to be different between groups (decisional conflict: *P* = 0.68; appropriate use of pharmacotherapy: *P* = 0.62; asthma control: *P* = 0.85).

**Table 2:** Two-month within- and between-group changes in asthma knowledge, decisional conflict, appropriate use of pharmacotherapy and asthma control (*N* = 51).

		Within-group change		Between-group change	
		MD [95% CI]	*P *value^a^	MD [95% CI]	*P *value^b^
**Knowledge of asthma** (QCALF score: a higher score is better)	Education + DA, n = 26	3.51 [1.74–5.3]	0.0002	1.50 [-1.03–4.0]	0.24
	Education alone, n = 25	2.02 [0.21–3.8]	0.03		
**Decisional conflict**^c^ (DCS score: a lower score is better)	Education + DA, n = 26	-2.93 [-5.38–-1.60]	0.0073	-1.25 [-2.97–1.90]	0.68
	Education alone, n = 25	-2.35 [-4.36–-1.27]	0.0093		
**Asthma control** (ACSS score: a higher score is better)	Education + DA, n = 26	8.47 [2.51–14.4]	0.0063	-0.79 [-9.31–7.7]	0.85
	Education alone, n = 25	9.26 [3.17–15.3]	0.0036		
		**PR [95% CI]**	***P *value**^**a**^	**PR [95% CI]**	***P *value**^**b**^
**Appropriate use of pharmacotherapy**	Education + DA, n = 26	1.35 [0.80–2.27]	0.25	0.81 [0.34–1.90]	0.62
	Education alone, n = 25	1.67 [0.84–3.30]	0.14		

ACSS: *Asthma Control Scoring System*; CI: Confidence interval; DCS: *Decisional Conflict Scale*; MD: Mean difference; PR: Prevalence ratio; QCALF: *Questionnaire de connaissances sur l’asthme de langue française*.

^a^We assessed whether changes in QCALF scores, DCS scores, ACSS scores, and in the proportions of appropriate users were different over time within each group.

^b^We assessed whether or not within-group changes in QCALF scores, DCS scores, ACSS scores, and proportions of appropriate users over time were different between groups.

^c^DCS scores underwent a natural log transformation. To facilitate interpretation, differences in means and their 95% CI were back-transformed on their original scale.

### 3.5 Ancillary analyses

We explored the impact of adding the DA to patient education on each of the five dimensions measured by the DCS. Results are displayed in Table 3. Between the two time points, participants of the education + DA group decreased their score on the uncertainty subscale from 8.4 [4.1–17.3] to 2.3 [1.2–4.4] (*P =* 0.0007). When compared to education alone, the addition of the DA was compatible with a further positive improvement (between-group MD: -2.76, 95% IC: [-7.69–1.01], *P =* 0.052).

**Table 3:** Two-month within- and between-group changes in scores on the informed, values clarity, support, uncertainty, and effective decision subscales of the DCS (*N* = 51).

DCS subscale^a^ (a lower score is better)	Group	Mean [95% CI] at baseline	Mean [95% CI] at 2-month follow-up	Within group MD [95% CI]	*P *value^b^	Between-group MD [95% CI]	*P *value^c^
Informed	Education + DA, n = 26	7.1 [3.6–13.8]	3.5 [1.9–6.6]	-2.01 [-4.90–1.21]	0.12	1.80 [-1.98–6.42]	0.36
	Education alone, n = 25	13.3 [6.7–26.3]	3.7 [1.9–7.0]	-3.62 [-8.99–-1.46]	0.007		
Values clarity	Education + DA, n = 26	7.6 [3.9–14.9[	3.8 [1.9–7.4]	-2.02 [-4.54–1.11]	0.09	1.33 [-2.40–4.22]	0.62
	Education alone, n = 25	12.9 [6.5–25.5]	4.8 [2.4–9.5]	-2.68 [-6.12–-1.17]	0.02		
Support	Education + DA, n = 26	12.0 [6.6–21.9]	4.1 [2.1–7.9]	-2.97 [-6.37–-1.39]	0.006	-1.30 [-3.87–2.28]	0.63
	Education alone, n = 25	12.4 [6.8–22.9]	5.4 [2.8–10.8]	-2.28 [—4.97–-1.05]	0.038		
Uncertainty	Education + DA, n = 26	8.4 [4.1–17.3]	2.3 [1.2–4.4]	-3.63 [-7.43–-1.77]	0.0007	-2.76 [-7.69–1.01]	0.052
	Education alone, n = 25	6.4 [3.1–13.2]	4.8 [2.5–9.2]	-1.31 [-2.73–1.58]	0.46		
Effective decision	Education + DA, n = 26	7.6 [3.9–15.0]	2.7 [1.5–5.0]	-2.79 [-4.93–-1.57]	0.0007	-1.56 [-3.53–1.45]	0.28
	Education alone, n = 25	6.0 [3.0–12.0]	3.4 [1.8–6.2]	-1.79 [-3.20–1.00]	0.051		

CI: Confidence interval; DCS: *Decisional Conflict Scale*.

^a^All DCS subscale scores underwent a natural log transformation.To facilitate interpretation, mean scores and their 95% CI were back-transformed on their original scale.

^b^We assessed whether changes in DCS subscale score were different over time within group.

^c^We assessed whether changes in DCS subscale score over time were different between groups.

## 4 Discussion

### 4.1 Key findings

We assessed whether or not adding a DA to patient education improved knowledge, and explored whether or not it lessened decisional conflict, and enhanced appropriate use of pharmacotherapy as well as asthma control among adults with asthma. In both groups, we found that improvements in asthma knowledge, decisional conflict, and asthma control occurred between the two time points. However, our results indicated that providing adults with asthma with the DA in addition to standard patient education did not result in further positive enhancements. These results lead us to make six observations.

First, patient education significantly increased knowledge among adults with asthma, as shown in previous trials [59–62], and was shown to have a positive impact on asthma control, along with evidence drawn from meta-analysis [7]. Based on asthma guidelines, empirical literature, and patient, health care professional and expert input, the logic model of asthma care can help better understand our results, as it underlines the role of asthma educators in influencing patients’ self-management skills, and in providing persons with asthma with feedback on health outcomes and behaviors [63]. In turn, patient education allows patients to better understand and manage their condition, and to achieve their optimal health potential [63].

Second, along with results from systematic reviews of interventions for enhancing adherence among patients with asthma [64, 65], patient education did not improve the appropriate use of pharmacotherapy, defined by a set of 11 hierarchical criteria [42] in the present study. In contrast, medication adherence scores, as measured by the *Self-Reported Medication-Taking Scale* [66], increased significantly with time in a randomized clinical trial assessing the impact of an asthma educational program, compared to usual care [62]. As the components of the educational program were quite similar to those of the present education intervention, we might hypothesize that the *Self-Reported Medication-Taking Scale* could have been more responsive to change [66] than the instrument used in the present study, and which was notably described as stringent [42].

Third, patient education was shown to have a beneficial impact on decisional conflict, especially on the informed and values clarity subscales of the DCS. To the best of our knowledge, our team was the first to assess whether or not patient education had an impact upon decisional conflict in asthma patients. We believe that patient education–by relying on open-ended questions to elicit patient illness and treatment beliefs and by providing patients with feedback [63]–helped participants gain knowledge about inhaled controller medications and encouraged them to communicate their preferences and concerns. In turn, the intervention might have addressed two modifiable factors that contribute to decisional conflict: feeling uniformed and unclear about personal values in making decisions [54].

Fourth, our results indicated that the addition of a DA to patient education did not bring further positive improvements in any of our pre-specified primary and secondary outcomes. In regards to knowledge and decisional conflict, our results contrast with the findings of a Cochrane systematic review and meta-analysis [16]. These findings showed that DAs improve informed and value-based decisions in patients facing either screening or treatment decisions, in comparison to usual care [16]. The randomized controlled trials that were included in this review assessed knowledge and decisional conflict soon after the patient’s exposure to decision aids [16]. In contrary, we measured these outcomes at two-month follow-up. We, therefore, believe that our DA, compared to patient education, might have a further positive effect on shorter-term outcomes, especially on decisional outcomes that would have been measured immediately after patient education. However, this hypothesis requires further investigation.

Fifth, to the best of our knowledge, whether or not adding a DA to nurse-led patient education improved knowledge, decisional conflict, appropriate use of asthma medications, and asthma control has not been previously assessed among adults with asthma. Again, based on the logic model of asthma care [63], we believe that asthma educators, by eliciting patient illness beliefs, and by providing patients with information and feedback on their self-management behaviors, helped patients acquire the knowledge, skills, and attitudes that were necessary to achieve their optimal health potential, and contributed to the collaborative participation that our DA was meant to foster more significantly than the DA itself. Nevertheless, educators reported that our DA helped them in eliciting participants’ concerns about inhaled controller medication use, and facilitated communication [30]. Although our study results showed that the addition of our DA did not result in additional improvements in decisional, behavioral, and health outcomes, previous qualitative results [30] suggested that it could fulfill a need for improved patient-clinician conversation [67]. Hence, our DA could have a positive impact on decision process outcomes, as described by the framework for measurement of shared decision making [40]. However, this hypothesis requires further investigation.

Sixth, we found that participants of the education + DA group significantly decreased their score on the uncertainty subscale, and the difference between groups was compatible with a further positive enhancement. We believe that our DA–by explicitly stating the decision to discuss and asking patients to indicate whether they would take the prescribed inhaled controller medication–might have a further positive effect on the state of being certain about decision, a predictor of decisional conflict, but the limited sample size may have hampered statistical significance in the present study.

### 4.2 Strengths and weaknesses

Our study has strengths. We used a sound methodology to compare our education alone and education + DA interventions, relying on randomization, blinding of assessor, and statistical models that accounted for repeated measurements. Nevertheless, our study had few limitations. First, the educators who were responsible for provision of patient education in both groups were not blinded to the experimental intervention and may have been more motivated to support control participants in making decisions. This may have diminished the impact of our DA on decisional conflict as well as reduced the probability to detect between-group differences, as reported elsewhere [68–70]. Second, to measure asthma control, we could have used either the *Asthma Control Questionnaire* [71, 72] or the *Asthma Control Test* [73, 74], for which the minimally important differences had already been estimated. Nevertheless, we chose to use the ACSS, because it not only has been validated in an asthma population recruited within the same setting [43], but it also fulfills the *Global Initiative for Asthma* criteria for assessment of asthma control [5]. Third, in the present study, carried out as part of the Master’s thesis of the first author (MG), the impact of adding the DA to patient education was evaluated among individuals diagnosed with asthma. These participants had good knowledge of asthma and did not have meaningful decisional conflict at the baseline visit. In the future, it would be interesting to assess the impact of a single or multiple exposures to our DA in a population of asthma patients, who misunderstand the role of ICS, and who have concerns about the use of the treatment. These patients might benefit more from the decision aid than the participants of this present study [30]. Though, this requires further investigation. Fourth, to our knowledge, our team developed the first DA to address ICS underuse in asthma and to assess whether or not adding such a DA to asthma education could enhance decisional, behavioral, and health outcomes, when compared to education alone. Further studies, powered to detect a group-by-time interaction of <5 points (that is, a smaller effect size), could be needed. Fifth, an explanatory sequential mixed methods study could have helped better explain our study results, because it would have allowed us to gain insights into the active components of education encounters and of DA that are effective in improving decisional, behavioral, and health outcomes [75]. Further studies using this methodology are needed.

### 4.3 Generalization

Because the participants were not recruited in primary care clinics and were followed by a certified asthma educator, our study sample may not represent the larger population of adults with mild to severe asthma to which the DA was first targeted [30]. Therefore, our results can be generalized to patients with mild to severe asthma who have access to a certified asthma educator.

## 5 Conclusions

In this study, we assessed the impact of adding a DA for adults with asthma considering the use of inhaled corticosteroids, with or without long-acting β_2_-agonists, to optimize asthma control. We found that patient education improves knowledge of asthma, decisional conflict, and asthma control whether our DA is added or not. Our DA may be useful to support less experienced educators in better structuring their educational interventions, because it helps guide discussions about inhaled controller medication use, which is a cornerstone of the asthma treatment regimen. To achieve optimal health status, the asthma treatment plan requires patients to adhere to several other measures [5]. Those requiring values-based decisions could be targeted by other DAs.

## Supporting Information

S1 File Study Protocol. We present the trial study protocol, that is: the complete and detailed plan for the conduct and analysis of the trial that the Institutional Ethics Committee of the Quebec Heart and Lung Institute approved before the trial began (approval number: CER20858).
(PDF)

S2 File CONSORT checklist. We present the completed CONSORT 2010 checklist that ensures the adequate reporting of our randomized trial.
(PDF)

S3 File Data set. We present the data set underlying the findings of our study (referred to as CER20858).
(SAS7BDAT)

S4 File Codebook. We present the codebook that describes the data set underlying the findings of our study (referred to as CER20858).
(PDF)
