# Supplementary material for: Impact of Adding a Decision Aid to Patient Education in Adults with Asthma: A Randomized Clinical Trial
Source: PLoS One. 2017 Jan 20;12(1):e0170055. doi: 10.1371/journal.pone.0170055 (PMC5249233; doi:10.1371/journal.pone.0170055)
Supplement: S1 File — We present the trial study protocol, that is: the complete and detailed plan for the conduct and analysis of the trial that the Institutional Ethics Committee of the Quebec Heart and Lung Institute approved before the trial began (approval number: CER20858). (PDF) [file pone.0170055.s001.pdf]

Project: **Development and Assessment of Shared Decision Making Aids in Asthma**

Authors:

Myriam Gagné, BEd [1]
France Légaré, MD PhD CCMF FCMF [2]
Jocelyne Moisan, PhD [3]
Louis-Philippe Boulet, MD FRCPC FCCP [1]

From:

[1] Chaire de transfert de connaissances, éducation et prévention en santé cardiovasculaire et respiratoire
Centre de recherche de l'Institut universitaire de cardiologie et de pneumologie de Québec
2725, chemin Sainte-Foy, Québec (Québec) Canada, G1V 4G5

[2] Chaire du Canada en implantation de la prise de décision partagée dans les soins primaires
Centre de recherche du CHU de Québec
Axe transfert des connaissances et évaluation des technologies et modes d'intervention en santé
Hôpital St-François d'Assise
10 rue Espinay, Québec (QC) Canada, G1L 3L5

[3] Chaire sur l'adhésion aux traitements de l'Université Laval et Axe Santé des populations et pratiques optimales en santé
Centre de recherche du CHU de Québec
Hôpital du St-Sacrement
1050 chemin Ste-Foy, Québec (QC) Canada, G1S 4L8

Corresponding Author:

Docteur Louis-Philippe Boulet
Chaire de transfert de connaissances, éducation et prévention en santé cardiovasculaire et respiratoire
Institut universitaire de cardiologie et de pneumologie de Québec (IUCPQ)
2725, chemin Sainte-Foy, Québec (Québec) Canada, G1V 4G5
Tél. : 418-656-4747
Courriel : lpboulet@med.ulaval.ca

Sponsors:

AllerGen NCE Inc.

Abstract

INTRODUCTION: Asthma is too often poorly controlled, resulting in a high socioeconomic burden. Although Canadian guidelines have suggested an optimal asthma management plan, which has been shown to improve asthma control, many patients do not follow physicians' recommendations and, particularly, do not take their medication as prescribed. Indeed, maintenance medication often remains underused. Non-compliance to asthma pharmacotherapy may be associated with poor patient-practitioner communication. In order to improve this and, by doing so, to lessen decisional conflict, shared decision making (SDM) aids may be effective. The Ottawa Decision Support Framework (ODSF) is intended for the development of SDM aids. It uses a three-step decision process: 1) identifying decision support needs, 2) developing, and 3) assessing aids. In asthma, a previous study, aiming at carrying out the first step of the ODSF, showed that decisional conflict was frequent among patients with asthma. Thus, it highlighted the need to develop SDM aids in asthma.

OBJECTIVES: To develop SDM aids in asthma. To assess their effect on asthma knowledge, decisional conflict, compliance with recommended use of pharmacotherapy, and asthma control among patients with mild to severe asthma. SDM aids are hypothesized to enhance knowledge and lessen decisional conflict. Consequently, they may also improve compliance with treatments, and optimize asthma control.

METHODS: The aids will be designed according to the International Patient Decision Aid Standards. They will also be refined in order to meet end-users' needs, which will be assessed by a questionnaire. Afterward, the aids will be assessed. To do so, 50 patients with mild to severe asthma will be enrolled in a pilot randomised controlled trial. Validated questionnaires will be used at baseline to assess outcome variables (knowledge, decisional conflict, compliance, and asthma control) and covariates (asthma knowledge, age, body mass index, atopy, environmental triggers, and respiratory tract infections). Enrolled patients will be randomized to either the experimental group, which will use the SDM aids, or to the control group (usual care). After a 2-month follow-up, outcome measurement will be undertaken to evaluate whether or not asthma knowledge enhances, decisional conflict lessens, and whether or not compliance with treatments and asthma control improve with the use of the aids. Generalised linear models will be used to assess the effect of the SDM aids on health outcomes. Models will adjust for covariates, if relevant.

PERSPECTIVES: The appropriate use of SDM aids may enhance asthma knowledge and lessen decisional conflict, which, in turn, may improve compliance with recommended use of pharmacotherapy and asthma control. Moreover, this study may provide patients and health professionals with a new and efficient approach to make informed, evidence-based and value-based decisions in the context of asthma management.

Introduction (background)

Asthma

Asthma is a chronic inflammatory condition affecting the lower airways. It is characterized by symptoms of breathlessness, chest tightness, wheezing and cough, as well as by airway obstruction which can be reversible or not (1).

Burden of asthma

In 2004, about 300 million people worldwide were suffering from asthma (2). In the past decade, the prevalence of this chronic inflammatory disease has undoubtedly increased, and asthma could affect up to 400 million people by 2025 (2). In Canada, its prevalence is among the highest worldwide (2), affecting more than 10% of the population (2) and making it the most common respiratory disease in the country (1).

According to Canadian Guidelines, asthma control is defined by the frequency of daytime (<4 days/week) and night time symptoms (<1 night/week), by the capacity to perform normal physical activity, by the degree and frequency of exacerbations (mild, infrequent), and by the frequency of rescue medication needs (<4 doses/week). Moreover, there should be no absenteeism from school or work due to asthma. The values of forced expiratory volume in 1 second (FEV_1, ≥90% personal best) and peak expiratory flow diurnal variation (PEF, <10%-15%) are also used to assess asthma control (3).

Asthma is unfortunately too often poorly controlled, resulting in a high socioeconomic burden (4). In Canada, hospital admissions account for the highest proportion of direct asthma-related costs from a societal perspective (5). Asthma indirect costs, such as work-loss days and premature deaths, are also important in the country (5).

Since the prevalence of asthma is increasing (2), and since costs of asthma increase with its severity and its poor management (6), developing new and efficient approaches to optimize asthma control is necessary.

Improving asthma control

Based on research information and effectiveness of treatment studies, Canadian Guidelines have suggested an optimal asthma management plan, which has been shown, when applied, to improve asthma control (1;7). However, many patients with asthma do not adhere to medical recommendations (4).

Adherence to treatments has first been defined as “the extent to which a person’s behaviour coincides with medical or health advice” (8). In addition, there are three components of adherence: acceptance, compliance and persistence (9). Compliance is “the extent to which a patient acts in accordance with prescribed interval and dose of a dosing regimen” (10) in order to optimize medication intake.

In asthma, both the insufficient use of controller medication and the overuse of rescue medication lead to uncontrolled asthma (11). Poor adherence with anti-inflammatory medication has indeed been shown in observational studies (12-14).

Poor adherence follows different patterns (15). Non-adherence may be unintentional, since patients may simply misunderstand their medication regimen. On the other hand, non-adherence may also be intentional, when patients worry about their medication intake and thereby decide not to fill their prescription or discontinue their treatment. In that case, poor compliance with asthma management plan is associated, among other factors, with patients' fears, misconceptions, and misunderstanding about treatments (4;16). In fact, communications are often suboptimal between patients and caregivers, who too often discuss neither patients' concerns about prescribed medication nor the pros and cons of the various recommendations (4). As a result of this, patients have to cope with a decisional conflict, which is defined as "the uncertainty about which course of action to take when choice among competing actions involves risk, loss, regret or challenge to personal life values" (17).

In a previous cross-sectional study, Des Cormiers and colleagues (Des Cormiers A, Legare F, Boulet LP. Assessment of Decisional Conflict in Asthma Management. *In preparation.*) assessed decisional conflict among 50 patients, aged 18 to 65 years, with mild to moderate, controlled or slightly uncontrolled asthma. These patients were recruited when attending the Outpatient Asthma Clinic of the *Institut universitaire de cardiologie et de pneumologie de Québec* (IUCPQ) or the IUCPQ Research Center. They were asked to fill the French version of the Decisional Conflict Scale (18). As a result of this, the study showed that 72% of enrolled patients had a meaningful decisional conflict score, defined as a total score greater than 2 on the decisional conflict scale. In other words, the study highlighted that 72% of enrolled patients with asthma were not comfortable with one or several decisions taken in their asthma management plan.

Lessening decisional conflict to optimize asthma control

Hence, developing a new and effective approach to asthma management that may enhance communication between caregivers and patients, enhance knowledge, lessen decisional conflict, foster the appropriate use of medication, and improve asthma control is of necessity.

State of knowledge

Since asthma control relies on adherence to pharmacotherapy, and since the appropriate use of medication depends on the patients' acceptance of the drug treatment plan, we make the assumption that if the use of shared decision making aids succeeds in enhancing knowledge and lowering decisional conflict, there may be an improvement of the patients' medication adherence, which, in turn, may lead to a better asthma control.

Achieving asthma control

In order to achieve asthma control, patients with asthma must avoid triggers and self-manage their symptoms, following a written action plan that physicians should have provided them with (3). Drug therapy is also an important part of asthma management, which highlights the

importance of physicians' adherence to asthma prescribing guidelines. Inhaled corticosteroids (ICS), as regular controller therapy, have been shown to effectively reduce asthma symptoms and improve, among other things, health-related quality of life (3). Long-acting beta$_2$-agonists (LABAs), leukotriene receptor antagonists (LTRAs), and, in severe asthma, oral corticosteroids could be required as add-on therapy if asthma control is not achieved with the use of ICS alone (3). Treating acute asthma symptoms relies on reliever therapy, such as inhaled fast-acting beta$_2$-agonists (FABAs). These, however, should be used at low-dose and low-frequency (3).

Several reasons may explain uncontrolled asthma. Physicians' non-adherence to asthma prescribing guidelines may be one of them. On the other hand, patients' lack of knowledge about the disease and its treatments may affect self-management (1). Moreover, inadequate environmental control at home, school, or work may lead to uncontrolled asthma, since atopy, tobacco smoke, and respiratory tract infections worsen symptoms (3). In addition, age is a predictor of the level of asthma control, since comorbidities increase with it (1). New evidences suggest that obesity may also be associated with uncontrolled asthma (1).

Other reasons may explain suboptimal asthma control. The level of asthma control may be over- or underestimated if an objective measurement of the patients' airflow obstruction is not undertaken (11). Moreover, many patients with asthma misunderstand their condition or the side-effects of pharmacotherapy (4), which highlights the need for educational interventions (11). Furthermore, both the insufficient use of controller medication and the overuse of reliever therapy, which underscore non-adherence, lead to uncontrolled asthma [1].

Achieving adherence to pharmacotherapy by lessening decisional conflict with the use of shared decision making aids

The appropriate use of asthma drugs is a critical issue. In asthma, patients' knowledge of medication and perception of health and drug costs have been shown as predictors of the appropriate use of pharmacotherapy (19). Having consulted a specialist for asthma seems also to modulate adherence to treatments (19). Age has also been shown as a predictor of compliance with medication (20).

A recent Cochrane review has highlighted interventions that aimed to improve adherence to medication in asthma (21). However, no intervention included in that Cochrane review relied on shared decision making, even though the approach has been shown to improve knowledge (22), which is a predictor of the appropriate use of pharmacotherapy (19). Four characteristics define shared decision making in a context of treatment (23): 1) caregiver's and patient's involvement; 2) values and expectations, relevant and evidence-based information; 3) discussion; and 4) agreement.

Decision aids are frequently used in shared decision making. They are defined as "evidence-based tools to prepare people to participate in making specific and deliberated choices among healthcare options in ways they prefer. They supplement (not replace) clinician's counselling" (24). Decision aids are educational (23). They provide detailed and evidence-based information

on the disease and its treatment options so that patients make informed decisions (24). They present risks, benefits and uncertainties of each treatment option in order to ensure that patients fully understand the ins and outs of the decision to be taken (24), which is easier when numbers, rather than words, are used to express probabilities (22). In addition, decision aids are designed to help patients clarify their values regarding the expected benefits of a treatment or its potentially undesirable consequences, so that patients' decisions are value-based (24). Consequently, decision aids lessen decisional conflict (22), which, otherwise, increases with the lack of support, resources, skills, self-confidence, and knowledge about treatment options, but also with social pressure, patients' unrealistic expectations, unclear values, and perception of others (25).

If shared decision making lowers decisional conflict (22), it also aims to optimize pharmacotherapy, bringing patients to adequately use their medication, so that they do not over- or underuse their drugs. In order to evaluate the effect of shared decision making on adherence to medication and health outcomes in asthma, Wilson and colleagues have enrolled 612 patients with poorly controlled asthma in a randomized controlled trial (26). Their study aimed to compare a shared decision making group to a clinician decision making group. After a 1-year follow-up, shared decision making has been shown to improve adherence to controller medication, lower the use of rescue medication, enhance quality of life and likelihood of asthma control, and reduce the number of medical visits. To our knowledge, this is the only study that has assessed shared decision making in asthma.

Even though shared decision making has been shown to lessen decisional conflict among patients who otherwise felt uninformed or unclear about their values (22), the question remains whether it lowers decisional conflict among patients with asthma. Moreover, the intervention assessed by Wilson and colleagues did not rely on shared decision making aids, but rather on the negotiation of decisions. In addition, although over 500 patient decision aids have already been developed worldwide (27), to our knowledge, none of those has been intended yet for asthma management.

The Ottawa Decision Support Framework (ODSF) is intended for the development of patient decision aids (24). It uses a three-step decision process: 1) identifying decision support needs, 2) developing, and 3) assessing decision aids. The framework has been developed according to psychosocial theories. It is based on the assumption that satisfied decisional needs lead to a better decision quality, which, in turn, enhances health behaviours and outcomes. According to the ODSF, improving decision quality relies on the use of decision aids.

Des Cormiers and colleagues have taken the first steps to develop shared decision making aids in asthma, based on the ODSF. Indeed, they carried out a decisional conflict evaluation among 50 patients with asthma and a needs assessment among 15 others (Des Cormiers A, Legare F, Boulet LP. Assessment of Decisional Conflict in Asthma Management. *In preparation.* & Des Cormiers A, Legare F, Boulet LP. Needs Assessment in Asthma Management. *In preparation.*) Those 15 patients were recruited at the ICUPQ Outpatient Asthma Clinic or at the IUCPQ

Research Center, and were asked to fill a French adapted version of the Population Needs Assessment Questionnaire (28). As a result of this cross-sectional study, Des Cormiers and colleagues identified which particular decisions regarding asthma management are problematic and for which it would be worth developing shared decision making aids. Consequently, since we believe that the acceptance of medication leads to the appropriate use of pharmacotherapy, and since the latter is known to improve asthma control, we aim, in the present study, to develop and then assess shared decision making aids in asthma.

Objectives

Based on a linear knowledge translation process, which makes the assumption that the appropriate use of shared decision making aids leads to medication acceptance and adherence, which, in turn, enhance asthma control, we aim to achieve the following objectives.

Primary objectives

- To develop shared decision making aids in asthma.
- Among adults with mild to severe asthma, to assess the effect of shared decision making aids on:
    - Asthma knowledge;
    - Patients' decisional conflict.

Secondary objectives

- Among adults with mild to severe asthma, to assess the effect of shared decision making aids on:
    - Compliance with recommended use of pharmacotherapy;
    - Asthma control.

Hypothesis

We hypothesize that our shared decision making aids will enhance knowledge and lessen decisional conflict. In addition, we believe that they may also improve compliance with recommended use of pharmacotherapy, by increasing maintenance drug use. Consequently, we consider that our shared decision making aids may lead to a better asthma control.

Methods

The aids will be developed, and then assessed.

Developing shared decision making aids

The shared decision making aids will be developed according to the International Patient Decision Aid Standards (IPDAS), a set of criteria that are internationally approved, and on which decision aids can be quality rated (27). The first aid to be developed will be about medication intake.

Developing prototypes of shared decision making aids

The aids will be designed in order to present a brief description of asthma and a summary of the current guidelines regarding asthma management. In addition, they will provide evidence-based information, such as probabilities of risks, benefits, and uncertainties associated with each treatment option, whenever possible (24). The aids will be designed in order to help patients clarify and express what matters the most to them, and to guide and facilitate discussion and communication of facts and values between patients and caregivers (24;27).

Gathering feedback on the aids and improving the aids

Once the aids will be designed, 2 health professionals (general practitioner, respirologist, or asthma educator) and 5 patients with asthma will be asked to provide feedback on the prototypes of the shared decision making aids. Patients will be recruited from the IUCPQ Outpatient Asthma Clinic by the asthma education nurse or from the IUCPQ Research Center by research nurses. Patients with a current diagnosis of asthma in their medical report will be eligible (see *Inclusion criteria* below). Relevant information on the study objectives will be given to patients interested to participate in the study by the study coordinator. Enrolled patients will sign an informed consent before participating in the study. The study will have been approved by the institutional ethics committee. According to the IPDAS criteria, none of the enrolled patients or health professionals reviewing the aids can further be involved in their field testing (27).

Enrolled patients and health professionals will have to fill an adapted version of the OHRI Acceptability Questionnaire (29). The questionnaire will have been given to them by the study coordinator. Feedback on the content, complexity, structure, schematisation, and length of the information provided in the aids will be sought, and suitability for decision making will be assessed. Once feedback will be gathered, the aids will be refined in order to meet end-users' needs. Afterward, 3 other health professionals and 5 other patients will fill the OHRI Acceptability Questionnaire to provide more feedback on the modified prototypes of the shared decision making aids. Once again, the aids will be refined according to their comments. Further feedback will be gathered if needed, until saturation is reached. Then, the aids will be evaluated.

Assessing shared decision making aids

A pilot study designed as a prospective randomized controlled trial will compare two groups of adults with mild to severe asthma, one in which decision aids are used (experimental group) and another in which the aids will not be used (control group). The effect of the aids on asthma knowledge, decisional conflict, compliance with recommended use of pharmacotherapy, and asthma control will be evaluated, because we make the assumption that the acceptance of medication may lead to the appropriate use of pharmacotherapy, which may, in turn, improve asthma control.

Evaluation of the aids

In order to recruit patients with mild to severe asthma in the study, medical reports of patients attending the IUCPQ Outpatient Asthma Clinic or having participated in studies at the Research

Center will be screened by the study coordinator. Patients who will meet the eligibility criteria (see *Inclusion criteria* below) will be contacted by the asthma nurse at the Outpatient Asthma Clinic prior to their meeting with her, or by the study coordinator if patients are recruited at the research center. Relevant information on the study objectives will be given by the study coordinator to eligible patients who will be interested to participate in the study. Those who will agree to participate in the study will sign an informed consent. The study will have been approved by the institutional ethics committee. Patients who will have given feedback on the aids in step 2 will not be enrolled, according to the IPDAS criteria.

The baseline visit (visit 1A) will take place at the IUCPQ Research Center and Outpatient Asthma Clinic. With the help of the study coordinator/asthma nurse, asthma control will be assessed, using the Asthma Control Scoring System (30). The study coordinator will evaluate patients' compliance with pharmacotherapy, using the Compliance Interview Questionnaire. In addition, the Decisional Conflict Scale (18) and the Asthma Knowledge Questionnaire (31) will be filled by patients. In order to control for covariates, if relevant, data on enrolled patients' health (age, height, weight, atopy, respiratory tract infections) and environmental factors will be gathered by the study coordinator, using the IUCPQ Asthma Consultation Form. For the same reason, enrolled patients' data on asthma knowledge at baseline will also be used. Visit 1A should last about 45 minutes.

After having filled the questionnaires, the study coordinator will give patients sealed envelopes in order to randomize them in the intervention or control group (see *Randomization (sequence concealment)* below). Consequently, patients will get, or not, the shared decision making aids, depending on the group they belong to (visit 1B). Those who will belong to the experimental group will have to pass through the steps of the aids before meeting the asthma nurse. Those belonging to the control group will skip this stage and therefore will meet the nurse directly, as in usual care. In both groups, there will be one meeting with the nurse per patient.

After a 2-month follow-up, patients will come to the IUCPQ Research Center for a 50-minute appointment (visit 2). With the help of the study coordinator, they will be asked to fill the same questionnaires as in the baseline visit.

Population, sample and study size

Target population

Men and women, between 18 and 65 years, with mild to severe asthma will be recruited. They must be able to read in order to adequately use the shared decision making aids, and understand the ins and outs of their treatment options.

Selection of subjects

Patients will be recruited among those attending the IUCPQ Outpatient Asthma Clinic or Research Center. Such convenience sampling will facilitate the enrolment of patients with mild to severe asthma. The inclusion criteria will be assessed through the patients' medical reports.

Inclusion criteria

- Eligible patients, aged 18 to 65 years, with a current diagnosis of mild to severe asthma will be recruited.
  - Asthma diagnosis will be made upon one of the following criteria (3):
    - FEV_1 increases by at least 12% after the use of a bronchodilator (and a minimum ≥200 mL);
    - Current asthma symptoms and a positive methacholine challenge test (20% fall in FEV_1 after inhalation of a provocative dose of methacholine 16 mg/mL);
    - A respirologist's current diagnosis of asthma found in the patient's medical report.
- Severity of asthma will be assessed according to prescribed pharmacotherapy, as suggested by current guidelines (1;32):
  - Patients with mild asthma using SABA as well as low doses (≤250 mcg/day beclomethasone or equivalent) of inhaled glucocorticosteroids;
  - Patients with moderate asthma using SABA as well as low to moderate doses (250mcg/day< dose ≤500 mcg/day beclomethasone or equivalent) of inhaled glucocorticosteroids, with or without additional therapy (LABA or LTRA);
  - Patients with severe asthma using high doses (>500 mcg/day beclomethasone or equivalent) of inhaled glucocorticosteroids, and additional pharmacotherapy (LABA, LTRA, or Prednisone), and SABA.

Exclusion criteria

- Patients having taken part in an asthma educational program at the IUCPQ Outpatient Asthma Clinic in the last 6 months;
- Patients aged 40 years or older with prebronchodilator FEV_1 <80% of predicted value (in order to ensure that patients with chronic obstructive pulmonary disease are excluded from the study);
- Smoking >10 pack-years.

Sample size

Since the study is a pilot, 50 patients are intended to be recruited to participate in the randomized control trial. Sample size calculations will be made during the study, based on gathered data. If feasible, more than 50 patients may be enrolled in order to get sufficient statistical power.

Randomization (sequence generation)

Using statistical software, a statistician will generate a blocked randomization list that will be used to allocate patients to either the intervention or the control group. Blocks of four will be used.

Randomization (sequence concealment)

Following the randomization list, 50 numbered opaque sealed and equally weighted envelopes will be given to the study coordinator. Each envelope will contain either the decision making aids (experimental group) or blank pages (control group). Allocation concealment will be

ensured since the study coordinator will have to give sequentially the sealed envelope to the enrolled patient who, once alone, will have to open it. Therefore, the study coordinator will not know in which group patients belong to.

Blinding

The person administering the questionnaires (study coordinator) will be blinded to the group assignment as will be the persons analysing the data (study coordinator and statistician).

Study intervention

In this study, the intervention is the use of shared decision making aids, which will be compared to usual care (not using the aids).

Shared decision making aids

The shared decision making aids that will be used in the intervention group will be the latest version of those developed in the previous stage of this study (developing shared decision making aids). Patients in the experimental group will receive the aids and will be asked to go through them prior to their meeting with the asthma education nurse at the IUCPQ Outpatient Asthma Clinic. During this meeting, the asthma nurse will review patient's written action plan. Individualized education and counselling will also be provided. The length of the meeting will be evaluated.

Usual care

In the control group, decision aids will not be used. As in usual care, patients meet directly the asthma nurse, who will review their written action plan, and provide individualized education and counselling. The length of the meeting will be evaluated.

Operational Definitions of Dependent Variables & Measurements

In this study, the dependent variables, or health outcomes, are asthma knowledge, decisional conflict, compliance with recommended use of pharmacotherapy, and asthma control. Covariates are asthma knowledge, age, body mass index, atopy, environmental triggers, and respiratory tract infections.

Asthma knowledge questionnaire (primary outcome)

Knowledge of asthma will be assessed by the Asthma Knowledge Questionnaire (31). The questionnaire will be filled by patients at baseline and after a 2-month follow-up. The French questionnaire comprises 37 items (true, false, don't know answers), evaluating 4 domains of asthma knowledge: biomedical, asthma severity, general knowledge and treatment (31). Developed in France, the original questionnaire had 38 items, but the eleventh has been withdrawn because it was not relevant in Quebec. The total knowledge score equals the good answers (rated 1) minus the wrong answers (rated -1). Don't know answers are rated 0. For each item, a negative score is indicative of asthma misunderstanding, a null score of a knowledge gap, and a positive score of gained asthma knowledge. The total knowledge score ranges from -37 to 37; thus, asthma knowledge will be modeled as a continuous variable. Psychometric properties of the questionnaire have been tested and showed good reliability and reproducibility (31).

Decisional conflict (primary outcome)

Decisional conflict will be assessed through a French version of OHRI Decisional Conflict Scale (18). The questionnaire will be filled at baseline and after a 2-month follow-up. Sixteen items evaluate 5 dimensions of decisional conflict (informed, values clarity, support, uncertainty, effective decision) using a Likert scale. Patients' responses range from 0 (strongly agree) to 4 (strongly disagree). Patients' responses are summed, divided by 16, and multiplied by 25. Consequently, the decisional conflict score ranges from 0 (low decisional conflict) to 100 (high decisional conflict). A score greater than 37.5 suggests that there is a meaningful decisional conflict, but a score that is less than 25 is not associated with it (33). The variable will nevertheless be analysed as a continuous one. The reliability of the OHRI Decisional Conflict Scale (English low literacy version) has been evaluated using a Cronbach's α, which value was above the 0.7 threshold (34). Test-retest coefficient also supports its reliability (35). The scale has been shown responsive to change, because it is able to detect an effect size of 0.2 to 0.3 in studies that compare an intervention group to a control group (33).

Compliance with treatments (secondary outcome)

Compliance with pharmacotherapy in the last week will be evaluated. An interview questionnaire based on the 2003 Asthma Consensus Conference guidelines (36) and adapted to the 2012 Asthma Consensus Conference guidelines (3) will be administered at baseline and after a 2-month follow-up. It has already been used to describe the appropriate use of asthma drugs among patients with moderate to severe asthma (19). The questionnaire is composed of a set of 11 hierarchical criteria; there were originally 12, but one of them is not relevant anymore. The set of criteria evaluates, in the last week, the use and the frequency of use of controller/rescue medication, and add-on therapy (if relevant). In order to be classified as compliant, patients need to meet all the 11 criteria (19), which set patients who are very compliant apart from others (37).

The questionnaire also allows compliance to maintenance/rescue/add-on medication to be expressed as a percent score ([doses taken/doses prescribed] × 100). In that case, a score that is ≥80% but ≤100% is indicative of compliance (38). The variable will nevertheless be analysed as a continuous one.

Asthma Control (Secondary Outcome)

Asthma control will be quantified using the Asthma Control Scoring System (ACSS) (39), which is based on Canadian Asthma Consensus Guidelines. The ACSS will be administered at baseline and after a 2-month follow-up. Two types of parameters are assessed by the ACSS: the frequency of symptoms in the last week (clinical parameter) and the percentage of predicted value of FEV_1 (physiological parameter). FEV_1 will be measured according to the ATS criteria (40) and predicted values will be obtained from IUCPQ. Both clinical and physiological parameters will be expressed as a percent score and the mean of those scores will give the global asthma control score. If a score ≥80% is indicative of asthma control, the variable will however be analysed as a continuous one. Discriminative properties of the ACSS have been evaluated (30). The reliability of the instrument has been assessed through internal consistency testing and test-retest, and

has been shown to be acceptable. Moreover, correlation with other instruments evaluated construct validity, which has been shown to be good. Furthermore, evaluative properties of the ACSS were assessed: responsiveness indexes supported its sensitivity, which means that the instrument detects changes over time and between groups.

Asthma knowledge (covariate)

Since the knowledge of asthma is also a predictor of decisional conflict and compliance with treatments, the values of the Asthma Knowledge Questionnaire (31), which will have been filled by patients at baseline, will be used. As a covariate, asthma knowledge will be modeled as a continuous variable.

Predictors of Asthma Control (Covariates)

Age (in years) and body mass index for adults (weight (kg) / [height (m)]^2 or weight (lb) / [height (in)]^2 x 703) will be assessed. In addition, inflammatory factors associated with asthma control will be measured. These factors are atopy (self-reported allergy; self-reported exposure to allergen), environmental triggers (smoking or exposure to second hand smoke), and respiratory tract infections in the last month. All those will be assessed through the IUCPQ Asthma Consultation Form. Covariate measurements will be undertaken at baseline. Questions regarding respiratory tract infections in the last month will also be asked after a 2-month follow-up. Factors associated with asthma control will be modeled as continuous or categorical variables.

Socio-demographic variables

Socio-demographic variables (gender, level of education – using the categories of the Institut de la statistique du Québec) and the year of asthma diagnosis will be measured in order to better describe the study population.

Data analysis

Descriptive analyses will be performed in order to describe the study population: continuous variables will be described as means or medians, depending on normality of distributions, and categorical variables as proportions.

Generalised linear models will be used to compare the intervention and control groups on the dependent variables (41). For asthma knowledge, decisional conflict, compliance with maintenance/rescue/add-on pharmacotherapy, and asthma control, a normal distribution and an identity link will be used, since all 4 variables are expressed as continuous ones. Assumptions of normality, and homoscedasticity will also be checked. A log transformation could be used to correct departure from normality, if relevant. For compliance with pharmacotherapy, a binomial distribution and a log link will be used as this variable is expressed as a binary one. In models, effects for group (intervention and control), time (baseline and 2-month follow-up) and group-by-time interaction will be included. The effect of the shared decision making aids on health outcomes will then be estimated by the interaction term between the group and time. Mean differences (continuous variables) and prevalence ratios (dichotomous variable) have been

chosen as effect measures. In addition, generalized estimating equations will be used in order to take repeated measures into account.

If covariates (asthma knowledge, age, body mass index, atopy, environmental triggers, and respiratory tract infections) are not distributed evenly between groups, we will adjust for them in analyses. Predictors of health outcomes will be incorporated into the model as confounders if they result in a >10% change in the effect measure, following a descending procedure. Exploratory subgroup analyses according to asthma severity will be conducted since pharmacotherapy and compliance with treatments are getting more complex with the severity of the disease. Statistical interaction of body mass index will also be exploratory assessed since this covariate is hypothesized to modify the effectiveness of intervention aiming at improving asthma control (42). In addition, exploratory statistical interaction of age, education and length of the patient's meeting with the asthma nurse will be evaluated since we hypothesize that the effect measure may vary according to the level of measurement of these variables. Regarding adherence to medication, sensitivity analyses will be carried out according to different cut-offs, that will range from 60 to 80% of inhaled prescribed doses.

SAS version 9.3 (SAS Institute Inc., Cary, USA) will be used to perform the statistical analyses, using PROC GENMOD. A 2-tailed *p*-value less than 0.05 will be considered indicative of statistical significance. Since the study is a pilot, power calculation will be made *a posteriori*. Alternatively, the study size is based on the number of patients that may realistically be recruited at the IUCPQ Research Center or Outpatient Asthma Clinic according to the timeframe. Nevertheless, sample size calculations will be made during the study, based on gathered data. If feasible, more patients may be enrolled in order to get sufficient statistical power.

Timeframe

Passing through the stages of this study will last 1 year. The intervention itself will be 2 months long, including baseline visit, follow-up, and outcome measurements.

Utility

Perspectives

The appropriate use of SDM aids may enhance knowledge of asthma and lessen decisional conflict, which, in turn, may improve compliance with recommended use of pharmacotherapy and asthma control. Moreover, this study may provide patients and health professionals with a new and efficient approach to make informed, evidence-based and value-based decisions in the context of asthma management.

The study conclusions will be the subject of a Master's thesis. They are also intended to be published in 2 peer-review articles. Moreover, gathered data on patients' compliance with pharmacotherapy are expected to be reanalysed in order to compare compliance to prescribed

pharmacotherapy and compliance to self-reported medication. Patients and health professionals' perception of the usability of the aids in an Outpatient Asthma Clinic is also expected to be further assessed in focus groups. In addition, patients' data on asthma knowledge may be used in order to validate a French Canadian Asthma Knowledge Questionnaire. Patients' data on decisional conflict and adherence may be used to further evaluate the concordance between patients' preferences and implemented options, according to the IPDAS criteria.

Bias

Convenience sampling and loss to follow-up may induce selection bias in this pilot randomised controlled trial. In order to avoid loss to follow-up, visit 2 will be scheduled at the end of visit 1. In addition, we will phone enrolled patients in the week preceding visit 2 in order to ensure that they remember their appointment at the IUCPQ Research Center.

Self-reported questionnaires are at risk of nondifferential misclassification that may weaken the effect of the aids on health issues. Although validated questionnaires are used in this study, they remain mostly subjective, and may induce information bias.

Furthermore, adherence to treatments is a complex phenomenon which has been linked to 200 different covariates (43). In addition, social pressure and lack of resources/ self-confidence are known to worsen decisional conflict (25) but will not be assessed in this study. Asthma control is also a complex phenomenon for which every single predictor will not have been assessed. Thus, residual confounding effect is possible.

Recent knowledge translation theories suggest that the knowledge translation process is more multidirectional than linear (44). Since our knowledge translation model is linear, it could not be excluded that external factors, such as health literacy and numeracy skills, may play a role in the success or failure of our intervention.

Generalizability

Assuming that internal validity is not threatened, the results of this study should be generalized to patients with mild to severe asthma, to those who are followed by a physician and by an asthma educator in a tertiary care center in Quebec City, and to those who are motivated by using shared decision making aids, and willing to do so.

Research Utility

If the effect of the aids on health outcomes is shown to be significantly positive, the aids may eventually be implemented in the Quebec health system, particularly through the Quebec Asthma and Chronic Obstructive Pulmonary Disease Network, and thereby may become key elements in the management of asthma.

References

Reference List

1. Lougheed MD, Lemiere C, Ducharme FM, Licskai C, Dell SD, Rowe BH, FitzGerald M, Leigh R, Watson W, Boulet LP. Canadian Thoracic Society 2012 guideline update: Diagnosis and management of asthma in preschoolers, children and adults. Can.Respir.J. 2012 Mar;19(2):127-64

2. Masoli M, Fabian D, Holt S, Beasley R. The global burden of asthma: executive summary of the GINA Dissemination Committee report. Allergy 2004 May;59(5):469-78

3. Lougheed MD, Lemiere C, Dell SD, Ducharme FM, FitzGerald JM, Leigh R, Licskai C, Rowe BH, Bowie D, Becker A, et al. Canadian Thoracic Society Asthma Management Continuum--2010 Consensus Summary for children six years of age and over, and adults. Can.Respir.J. 2010 Jan;17(1):15-24. PMCID:PMC2866209

4. FitzGerald JM, Boulet LP, McIvor RA, Zimmerman S, Chapman KR. Asthma control in Canada remains suboptimal: the Reality of Asthma Control (TRAC) study. Can.Respir.J. 2006 Jul;13(5):253-9. PMCID:PMC2683303

5. Ungar WJ, Coyte PC, Chapman KR, MacKeigan L. The patient level cost of asthma in adults in south central Ontario. Pharmacy Medication Monitoring Program Advisory Board. Can.Respir.J. 1998 Nov;5(6):463-71

6. Bahadori K, Doyle-Waters MM, Marra C, Lynd L, Alasaly K, Swiston J, FitzGerald JM. Economic burden of asthma: a systematic review. BMC.Pulm.Med. 2009;9:24. PMCID:PMC2698859

7. Bateman ED, Hurd SS, Barnes PJ, Bousquet J, Drazen JM, FitzGerald M, Gibson P, Ohta K, O'Byrne P, Pedersen SE, et al. Global strategy for asthma management and prevention: GINA executive summary. Eur.Respir.J. 2008 Jan;31(1):143-78

8. Haynes B, Taylor D, Sackett D. Compliance in Health Care. Baltimore: The John Hopkins University Press; 1979.

9. Wahl C, Gregoire JP, Teo K, Beaulieu M, Labelle S, Leduc B, Cochrane B, Lapointe L, Montague T. Concordance, compliance and adherence in healthcare: closing gaps and improving outcomes. Healthc.Q. 2005;8(1):65-70

10. Cramer JA, Roy A, Burrell A, Fairchild CJ, Fuldeore MJ, Ollendorf DA, Wong PK. Medication compliance and persistence: terminology and definitions. Value.Health 2008 Jan;11(1):44-7

11. Becker A, Lemiere C, Berube D, Boulet LP, Ducharme FM, FitzGerald M, Kovesi T. Summary of recommendations from the Canadian Asthma Consensus guidelines, 2003. CMAJ. 2005 Sep 13;173(6 Suppl):S3-11. PMCID:PMC1329945

12. Gamble J, Stevenson M, McClean E, Heaney LG. The prevalence of nonadherence in difficult asthma. Am.J.Respir.Crit Care Med. 2009 Nov 1;180(9):817-22

13. Krishnan JA, Riekert KA, McCoy JV, Stewart DY, Schmidt S, Chanmugam A, Hill P, Rand CS. Corticosteroid use after hospital discharge among high-risk adults with asthma. Am.J.Respir.Crit Care Med. 2004 Dec 15;170(12):1281-5

14. Stempel DA, Roberts CS, Stanford RH. Treatment patterns in the months prior to and after asthma-related emergency department visit. Chest 2004 Jul;126(1):75-80

15. Boulet LP, Vervloet D, Foster JM. Adherence: The Goal to Control Asthma (*In press*). Clin.Chest.Med. Forthcoming.

16. Boulet LP. Perception of the role and potential side effects of inhaled corticosteroids among asthmatic patients. Chest 1998 Mar;113(3):587-92

17. North American Nursing Diagnosis Association International. NANDA Nursing Diagnoses: Definitions & Classification 2005-2006. Philadelphia: NANDA International; 2005.

18. O'Connor AM. Decisional Conflict Scale [French Translation Legare et al 2009] [document on the Internet]. 2005. Available from: http://www.ohri.ca/decisionaid/ Date accessed: 5-4-2012.

19. Jobin MS, Moisan J, Bolduc Y, Dorval E, Boulet LP, Gregoire JP. Factors associated with the appropriate use of asthma drugs. Can.Respir.J. 2011 Mar;18(2):97-104. PMCID:PMC3084424

20. Lacasse Y, Archibald H, Ernst P, Boulet LP. Patterns and determinants of compliance with inhaled steroids in adults with asthma. Can.Respir.J. 2005 May;12(4):211-7

21. Haynes RB, Ackloo E, Sahota N, McDonald HP, Yao X. Interventions for enhancing medication adherence. Cochrane.Database.Syst.Rev. 2008;(2):CD000011

22. Stacey D, Bennett CL, Barry MJ, Col NF, Eden KB, Holmes-Rovner M, Llewellyn-Thomas H, Lyddiatt A, Legare F, Thomson R. Decision aids for people facing health treatment or screening decisions. Cochrane.Database.Syst.Rev. 2011;(10):CD001431

23. Charles C, Gafni A, Whelan T. Shared decision-making in the medical encounter: what does it mean? (or it takes at least two to tango). Soc.Sci.Med. 1997 Mar;44(5):681-92

24. O'Connor AM. Ottawa Decision Support Framework (ODSF) to Address Decisional Conflict [document on the Internet]. 2006. Available from: www.ohri.ca/decisionaid Date accessed: 5-4-2012.

25. O'Connor AM, Jacobsen MJ, Stacey D. An evidence-based approach to managing women's decisional conflict. J.Obstet.Gynecol.Neonatal Nurs. 2002 Sep;31(5):570-81

26. Wilson SR, Strub P, Buist AS, Knowles SB, Lavori PW, Lapidus J, Vollmer WM. Shared treatment decision making improves adherence and outcomes in poorly controlled asthma. Am.J.Respir.Crit Care Med. 2010 Mar 15;181(6):566-77. PMCID:PMC2841026

27. International Patient Decision Aid Standards (IPDAS) Collaboration. International Patient Decision Aid Standards [document on the Internet]. 2012. Available from: http://ipdas.ohri.ca/IPDAS_checklist.pdf Date accessed: 6-21-2012.

28. Jacobsen MJ, O'Connor AM. Population Needs Assessment [document on the Internet]. Ottawa: Ottawa Hospital Research Center; 2006. Available from: http://decisionaid.ohri.ca/docs/implement/Population_Needs.pdf Date accessed: 8-28-2012.

29. O'Connor AM, Cranney A. Sample Tool: Acceptability (Osteoporosis Therapy) [document on the Internet]. 1996. Available from: www.ohri.ca/decisionaid Date accessed: 5-4-2012.

30. LeBlanc A, Robichaud P, Lacasse Y, Boulet LP. Quantification of asthma control: validation of the Asthma Control Scoring System. Allergy 2007 Feb;62(2):120-5

31. Nguyen L, Raherison C, Bozonnat MC, Lheureux M, Nocent C, Tunon-de-Lara JM, Taytard A. [Validation of an asthma knowledge questionnaire]. Rev.Mal Respir. 2003 Dec;20(6 Pt 1):871-80

32. Boulet LP, Becker A, Berube D, Beveridge R, Ernst P. [Summary of the recommendations of the Canadian Consensus Conference on Asthma 1999. Canadian Asthma Consensus Group]. CMAJ. 1999 Nov 30;161(11 Suppl Resume):SF1-14. PMCID:PMC1230848

33. O'Connor AM. User Manual - Decisional Conflict Scale (16 item statement format) [document on the Internet]. Ottawa: Ottawa Hospital Research Institute; 2010. Available from: http://decisionaid.ohri.ca/docs/develop/User_Manuals/UM_Decisional_Conflict. Date accessed: 6-26-2012.

34. Scholl I, Koelewijn-van LM, Sepucha K, Elwyn G, Legare F, Harter M, Dirmaier J. Measurement of shared decision making - a review of instruments. Z.Evid.Fortbild.Qual.Gesundhwes. 2011;105(4):313-24

35. O'Connor AM. Validation of a decisional conflict scale. Med.Decis.Making 1995 Jan;15(1):25-30

36. Lemiere C, Bai T, Balter M, Bayliff C, Becker A, Boulet LP, Bowie D, Cartier A, Cave A, Chapman K, et al. Adult Asthma Consensus Guidelines update 2003. Can.Respir.J. 2004 May;11 Suppl A:9A-18A

37. Jobin MS. Facteurs associés à un usage approprié des médicaments contre l'asthme chez les 12 à 45 ans: Mémoire de maîtrise Québec: Université Laval; 2007. 1-151 p.

38. Cochrane GM. Compliance and outcomes in patients with asthma. Drugs 1996;52 Suppl 6:12-9

39. Boulet LP, Boulet V, Milot J. How should we quantify asthma control? A proposal. Chest 2002 Dec;122(6):2217-23

40. Miller MR, Hankinson J, Brusasco V, Burgos F, Casaburi R, Coates A, Crapo R, Enright P, van der Grinten CP, Gustafsson P, et al. Standardisation of spirometry. Eur.Respir.J. 2005 Aug;26(2):319-38

41. Vittinghoff E, Shiboski SC, Glidden DV, McCulloch CE. Repeated Mesures and Longitudinal Data Analysis [document on the Internet]. [10.1007/b138825]. New York: Springer; 2012. XV. Available from: http://www.springerlink.com/content/v78657240v787833/fulltext.pdf Date accessed: 7-4-2012.

42. Ayala E, Wilson SR, Ma J, Knowles SB, Buist AS, Strub P, Lavori PW. Influence of body mass index on effects of a shared asthma treatment decision-making intervention. Am.J.Respir.Crit Care Med. 2012 Mar 1;185(5):591-3

43. Vermeire E, Hearnshaw H, Van RP, Denekens J. Patient adherence to treatment: three decades of research. A comprehensive review. J.Clin.Pharm.Ther. 2001 Oct;26(5):331-42

44. Ward V, House A, Hamer S. Developing a framework for transferring knowledge into action: a thematic analysis of the literature. J.Health Serv.Res.Policy 2009 Jul;14(3):156-64. PMCID:PMC2933505

Annexe 1. Data analysis

Table 1: Dependent variable and measurements

	TYPE OF VARIABLE	MEASUREMENT	TYPE OF MEASUREMENT	DATA COLLECTION	
				BASELINE	2 MONTH
**Primary outcome**					
**Decisional Conflict**	Continuous	Decisional Conflict Scale	Subjective & self-reported	X	X
**Secondary outcomes**					
**Compliance with treatments**	Binary	Compliance Interview Questionnaire	Subjective & self-reported	X	X
**Compliance with controller/rescue/add-on pharmacotherapy**	Continuous	Compliance Interview Questionnaire	Subjective & self-reported	X	X
**Asthma Control**	Continuous	Asthma Control Scoring System	Clinical parameter: Subjective & self-reported<br>Physiological param.: Objective	X	X

Table 2: Covariates and measurements

VARIABLE	TYPE OF VARIABLE	MEASUREMENT	DATA COLLECTION	
			BASELINE	2 MONTH
**Associated with decisional conflict**				
Asthma Knowledge	Continuous	Asthma knowledge Questionnaire	X	
**Associated with compliance with treatments**				

Asthma knowledge	Continuous	Asthma knowledge Questionnaire	X	
Age	Continuous	IUCPQ Asthma Consultation Form		
**Associated with asthma control**				
Age	Continuous	IUCPQ Asthma Consultation Form	X	
Body mass index	Categorical	IUCPQ Asthma Consultation Form	X	
Environmental triggers (smoking/ second hand smoke)	Binary	IUCPQ Asthma Consultation Form	X	
Atopy (self-reported allergy/ allergen)	Binary	IUCPQ Asthma Consultation Form	X	
Respiratory tract infections in the last month	Binary	IUCPQ Asthma Consultation Form	X	X
Asthma knowledge	Continuous	Asthma knowledge Questionnaire	X	

Table 3: Comparative analysis of covariates at baseline

Covariate	Intervention group (shared decision making)	Control group (usual care)	
**Associated with decisional conflict**			
Asthma knowledge	X	X	Mean
**Associated with compliance with asthma treatments**			
Asthma knowledge	X	X	Mean
Age	X	X	Mean
**Associated with asthma control**			
Age	X	X	Mean
Body mass index (underweight, normal weight, overweight, obesity)	X	X	Propo
Environmental triggers (smoking/ second hand smoke))	X	X	Propo
Atopy (self-reported allergy/ allergen)	X	X	Propo
Respiratory tract infections in the last month	X	X	Propo
Asthma knowledge	X	X	Mean

Table 4: Comparative analysis of socio-demographic variables at baseline

Socio-demographic variable	Intervention group (shared decision making)	Control group (usual care)	
**Sex**			
**Sex**	X	X	Proportion
**Level of education**			
University degree	X	X	Proportion
**Years with asthma**	X	X	Mean (standard [illegible]

Table 5: Comparative analysis of dependent variables at baseline

Covariate	Intervention group (shared decision making)	Control group (usual care)	Test	P-value
**Primary outcomes**				
Decisional conflict	X	X	Student *t*-test	X
Asthma knowledge	X	X	Student *t*-test	X
**Secondary outcomes**				
Compliance with treatments	X	X	Pearson's chi-squared test	X
Compliance with:				
Controller medication	X	X	Student *t*-test	X
Rescue medication	X	X	Student *t*-test	X
Add-on therapy	X	X	Student *t*-test	X
Asthma control	X	X	Student *t*-test	X

Table 6: Effect* of shared decision making aids on dependent variables

Dependent variable	Baseline		2 month		Model	Distribution	Link	Group IG CG	Time T1 T2	Interaction term $G \times T$	[illegible]
	IG	CG	IG	CG							
**Primary outcomes**											
**Decisional conflict (DC)**	X	X	X	X	Generalised linear model	Normal	Identity	X	X	X	[illegible]
**Asthma knowledge**	X	X	X	X	Generalised linear model	Normal	Identity	X	X	X	[illegible]
**Secondary outcome**											
**Compliance with treatments**	X	X	X	X	Generalised linear model	Binomial	log	X	X	X	[illegible]
**Compliance with:**											
**Controller medication**	X	X	X	X	Generalised linear model	Normal	Identity	X	X	X	[illegible]
**Rescue medication**	X	X	X	X	Generalised linear mdl	Normal	Identity	X	X	X	[illegible]
**Compliance with:**											
**Add-on therapy**	X	X	X	X	Generalised linear model	Normal	Identity	X	X	X	[illegible]
**Asthma control**	X	X	X	X	Generalised linear model	Normal	Identity	X	X	X	[illegible]

IG: Intervention group (shared decision making) ***CG***: Control group (usual care) ***T1***: Baseline ***T2***: 2-month follow-up

* The effect of shared decision making aids on health outcomes will be estimated with their 95% confidence interval. The effect of the aids on asthma knowledge, decisional conflict, compliance with

controller/rescue/add-on pharmacotherapy, and asthma control will be expressed as means, since these variables are continuous. On the other hand, the effect of the aids on compliance with treatments (a binary variable) will be expressed as prevalence ratios. Both types of model will adjust for covariates, if a confounding variable results in a ≥10% change of the effect measure.

† P-value of the interaction term.
