# Supplementary material for: Impact of Adding a Decision Aid to Patient Education in Adults with Asthma: A Randomized Clinical Trial
Source: PLoS One. 2017 Jan 20;12(1):e0170055. doi: 10.1371/journal.pone.0170055 (PMC5249233; doi:10.1371/journal.pone.0170055)
Supplement: S4 File — We present the codebook that describes the data set underlying the findings of our study (referred to as CER20858). (PDF) [file pone.0170055.s004.pdf]

2016-12-22
Project title: Impact of Adding a Decision Aid to Patient Education in Adults with Asthma: a Randomized Clinical Trial
Principal investigator: Dr. Louis-Philippe Boulet
Study coordinator: Myriam E. Gagne
Institutional Ethics Committee approval number: CER20858

CODEBOOK

Data can be found in the following document: S3_data-set_cer20858.sas7bdat

Variable	Description	Values	Comments
**IDSUJET**	Unique identification number		<from 3001 to 3052>
**RANDO**	Randomization number		<from 0 to 52>
**INTERV**	Intervention	0. Education (control group)<br>1. Education + DA (experimental group)	
**ASEX**	Gender at baseline	1. Man<br>2. Woman	
**AAGE**	Age (years) at baseline		
**AIMC**	Body mass index, BMI (kg/m^2) at baseline		
**AIMC_CAT**	BMI at baseline	0. BMI<25<br>1. 25≤BMI<30<br>2. BMI≥30	
**AHIGHEST_EDUC**	Highest level of education attained at baseline	0. <University<br>1. University completed	
**AACSSVEMS_L**	Forced expiratory volume in one second, FEV_1 (L) at baseline		
**AACSSVEMS_PCT_GLI2012**	FEV_1 (% predicted) at baseline		
**AACSSCVF_L**	Forced vital capacity, FVC (L) at baseline		
**AACSSCVF_PCT_GLI2012**	FVC (% predicted) at baseline		
**ASEVERIT**	Asthma severity at baseline	0. Severe<br>1. Moderate<br>2. Mild	
**ANRX**	Number of asthma medications at baseline		<from 1 to 6>
**AASTH_DUR**	Duration of asthma (years) at baseline		<”.” if missing data>
**AHTAB**	Smoking status at baseline	0. Non-smoker<br>1. Ex-smoker<br>2. Current smoker	
**AATOPY**	Self-reported allergy at baseline	0. No<br>1. Yes	<”.” if missing data>

**AARTILM**	Self-reported respiratory tract infections at baseline	0. No<br>1. Yes	<”.” if missing data>
**AQCALF**	*Questionnaire de connaissances sur l’asthme de langue française* (QCALF) score at baseline		<from -37 to +37>
**ADCS_TOT**	*Decisional Conflict Scale* (DCS) total score at baseline		<from 0 to 100>
**ADCS_INFORMED**	DCS informed subscale score at baseline		<from 0 to 100>
**ADCS_VALUES**	DCS values clarity subscale score at baseline		<from 0 to 100>
**ADCS_SUPPORT**	DCS support subscale score at baseline		<from 0 to 100>
**ADCS_UNCERTAINTY**	DCS uncertainty subscale score at baseline		<from 0 to 100>
**ADCS_EFFECTIVE**	DCS effective decision subscale score at baseline		<from 0 to 100>
**ACIQ**	Appropriate medication use at baseline	0. No<br>1. Yes	
**AACSS**	*Asthma Control Scoring System* (ACSS) score at baseline		<from 20 to 100>
**BQCALF**	QCALF score at 2-month follow-up		<from -37 to +37><br><”.” if missing data>
**BDCS_TOT**	DCS total score at 2-month follow-up		<from 0 to 100><br><”.” if missing data>
**BDCS_INFORMED**	DCS informed subscale score at 2-month follow-up		<from 0 to 100><br><”.” if missing data>
**BDCS_VALUES**	DCS values clarity subscale score at 2-month follow-up		<from 0 to 100><br><”.” if missing data>
**BDCS_SUPPORT**	DCS support subscale score at 2-month follow-up		<from 0 to 100><br><”.” if missing data>
**BDCS_UNCERTAINTY**	DCS uncertainty subscale score at 2-month follow-up		<from 0 to 100><br><”.” if missing data>
**BDCS_EFFECTIVE**	DCS effective decision subscale score at 2-month follow-up		<from 0 to 100><br><”.” if missing data>
**BCIQ**	Appropriate medication use at 2-month follow-up	0. No<br>1. Yes	<”.” if missing data>
**BACSS**	ACSS score at 2-month follow-up		<from 20 to 100><br><”.” if missing data>
